# Focused Update on Randomized Trials in Acute Coronary Syndromes: A Scoping Review

**DOI:** 10.3390/jcm15218704

**Published:** 2026-11-07

**Authors:** Felicita Andreotti, Adhithya Narayanan Balaji, Luigi Cappannoli, Giovanni Gambassi, Adelaide Iervolino, Aldo Pietro Maggioni, Massimo Massetti, Francesco Burzotta

**Affiliations:** 1 Cardiovascular Science Department, Fondazione Policlinico Universitario Agostino Gemelli IRCCS, 00168 Rome, Italy; 2 Cardiothoracic Department, Catholic University of the Sacred Heart, 00168 Rome, Italy; 3 General Medicine and Aging Unit, Fondazione Policlinico Universitario Agostino Gemelli IRCCS, 00168 Rome, Italy; 4 Department of Medical and Surgical Sciences, Catholic University of the Sacred Heart, 00168 Rome, Italy; 5 Echocardiography Laboratory, Division of Cardiology, Massachusetts General Hospital, Harvard Medical School, Boston, MA 02114, USA; 6 Cardiovascular Pathophysiology and Therapeutics PhD Program, Federico II University Hospital, 80131 Naples, Italy; 7 Centro Studi ANMCO— Heart Care Foundation, 50121 Florence, Italy

**Keywords:** acute coronary syndrome, antiplatelet, beta-blocker, bleeding, death, myocardial infarction, randomized trial, revascularization

## Abstract

**Background and Aim:** Evidence-based medicine is dynamic. We appraised recent evidence on the management of acute coronary syndromes (ACS). **Methods:** A PRISMA scoping review of randomized controlled trials (RCTs) published in PubMed/MEDLINE between January 2025 and March 2026 was conducted. Only trials involving ≥500 ACS patient-years and reporting total or cardiovascular death, myocardial infarction, stroke and clinically relevant safety events were included. Comorbid atrial fibrillation and shock were excluded. Data extraction was prespecified. **Results:** Ten RCTs on antiplatelet therapy, percutaneous coronary intervention (PCI) and beta-blocker treatment were retained and analyzed for bias. Within the limits of each trial, the following messages emerge. After ACS-PCI, very early (day 4) antiplatelet monotherapy may not provide adequate anti-ischemic protection compared to 12-month dual antiplatelet therapy (DAPT; NEO-MINDSET trial). In ACS-PCI patients at low ischemic risk, 1-month DAPT followed by P2Y12 inhibitor alone can reduce major or clinically relevant nonmajor bleeding compared to 12-month DAPT, without apparent loss of anti-ischemic protection (TARGET-FIRST and 4D-ACS trials). For ACS patients receiving 12-month DAPT, ischemic and bleeding outcomes seem not to differ significantly when aspirin is given once or twice daily (ANDAMAN trial), nor when DAPT is clopidogrel-based or ticagrelor-based within specific populations (Canadian Bayesian pragmatic TC4 trial). In ACS patients undergoing coronary bypass, 12-month ticagrelor-based DAPT compared to aspirin alone enhances major bleeding risk without apparent anti-ischemic benefit (TACSI trial). Complete versus culprit-only revascularization leads to lower 3-year rates of ischemic events in older ACS patients with multivessel disease across renal function strata (FIRE trial). For ACS patients with multivessel disease, immediate complete revascularization failed to reach non-inferiority compared to pre-discharge complete revascularization (OPTION-STEMI trial). For patients with a left ventricular ejection fraction above or equal to 40% after myocardial infarction, beta-blocker treatment compared to none, over a follow-up ≥3.5 years, improved outcomes in BETAMI-DANBLOCK but not in REBOOT-CNIC. **Conclusions:** The data retained by this scoping review have implications that impact common clinical-practice decisions.

## 1. Introduction

In recent months, a plethora of studies have addressed various management strategies for patients with acute coronary syndromes (ACS), including the pre-hospital phases, the in-hospital triage process, the choice of antithrombotic therapies, the refinement of interventional procedures, and pre-discharge decisions on long-term treatment. With the aim of identifying and summarizing recent information relevant to the management of ACS patients, we conducted a Preferred Reporting Items for Systematic reviews and Meta-Analyses scoping review (PRISMA-ScR) of clinical outcome trials published between 1 January 2025 and 29 March 2026.

## 2. Materials and Methods

### 2.1. Search Strategy

The PRISMA extension for scoping reviews [1] and a prespecified—albeit not formally registered—protocol were followed. PubMed/MEDLINE databases were searched including the headings “acute coronary syndrome”, “acute myocardial infarction” and (publication year) “2025” or “2026”. Automatic tools were applied to identify randomized controlled trials (RCTs), patients ≥19 years of age, full-text articles, and English language. The initial search ended on 30 March 2026. Screening was conducted independently by two investigators (FA and ANB), excluding electronic publications prior to 2025 and retaining all relevant publications (issue or electronic or manually retrieved from citation searches) up to 29 March 2026. Disagreements were resolved by consensus or by input from a third investigator. Search details are provided in Supplementary Table S1. Ethics committee approval was not applicable given the anonymized nature of the search. The PRISMA-ScR checklist is provided in Supplementary Table S2.

### 2.2. Inclusion/Exclusion Criteria

The prespecified inclusion criteria were: (1) an ACS population; (2) randomized and parallel allocation of treatments; (3) sample follow-up of at least 500 patient-years; and (4) reporting of clinically relevant outcomes. The prespecified ≥500 patient-year threshold was chosen to focus on trials powered for major clinical outcomes.

Prespecified exclusion criteria were: (1) non-parallel treatments; (2) mixed (e.g., acute and chronic coronary syndrome) or super-selected populations (e.g., with small-vessel or in-stent restenosis lesions); (3) comorbid atrial fibrillation or cardiogenic shock; and (4) subgroup analyses that were *post hoc* or did not preserve the randomization factor.

### 2.3. Outcomes of Interest and Data Extraction

“Efficacy” endpoints were defined as clinically relevant if they included total or cardiovascular death, myocardial infarction (MI), or stroke. Safety endpoints of interest were those reported in each trial. Within the PICO (Population–Intervention–Comparator–Outcomes) framework, the following data were extracted using prespecified charts: trial name/design/funding; main clinical characteristics, including age, sex, and diagnosis; number of patients overall and in each arm; type of active and control treatment; follow-up duration; primary endpoint definition; safety endpoint definition; and main clinical findings.

### 2.4. Risk of Bias and Excluded Trials

Retained reports were assessed using the revised risk of bias tool [2]. Risk was classified as low, of some concern, or high with respect to randomization (or identification/recruitment for cluster randomization), deviations from intended interventions, missing outcomes, outcome measures, and selection of reported results. A log of excluded reports was kept as recommended by Cochrane, with reasons for exclusion [3].

## 3. Results

### 3.1. Search Results

**Figure 1:**
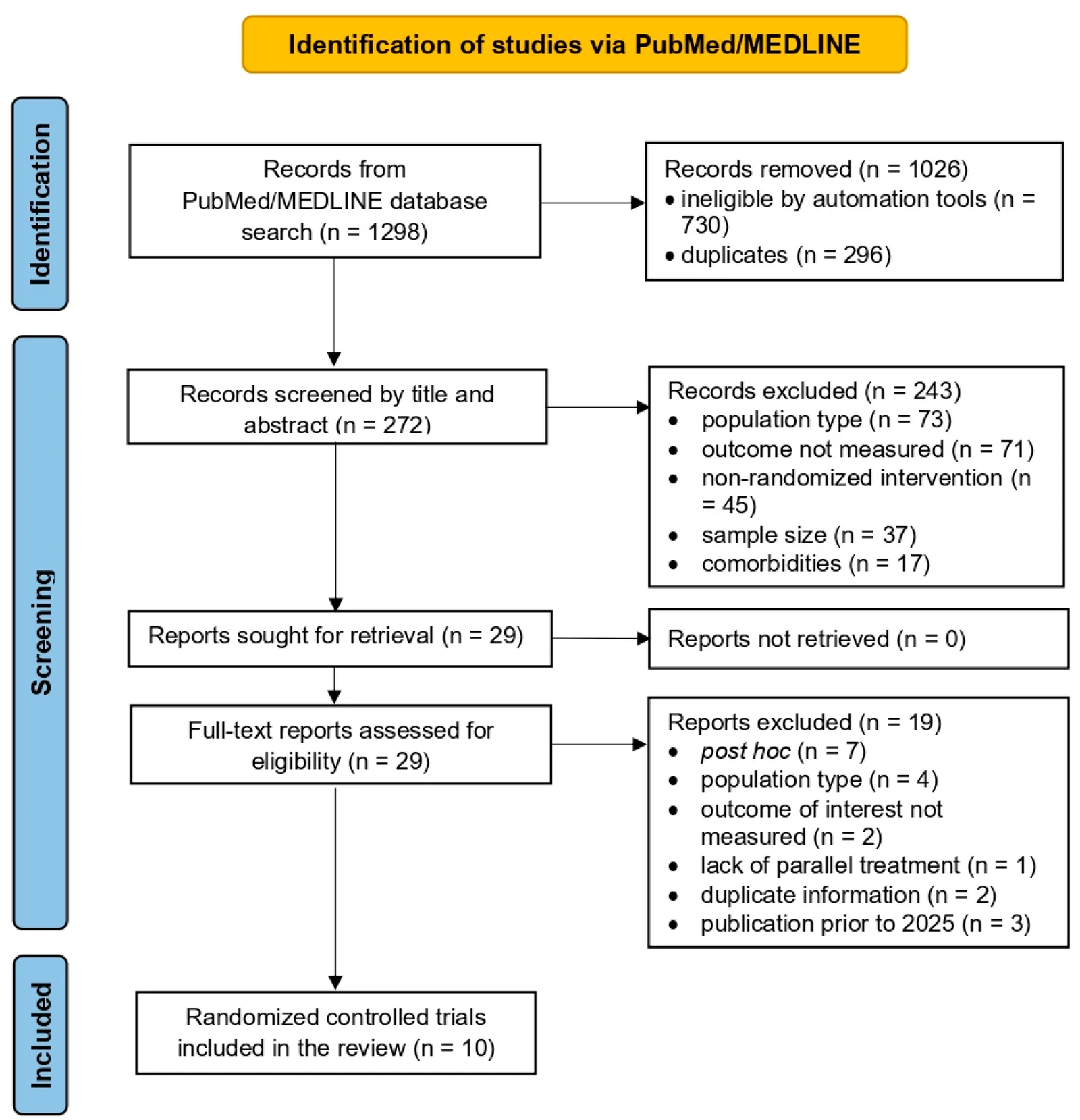
PRISMA-ScR study-selection flow chart. From 1298 records initially identified, 272 were screened and 10 reports finally retained.

The initial search terms identified 1298 records; removal of duplicates and ineligible records by automated tools resulted in 272 records screened on title and abstract; of these, 243 were excluded for type of patients, outcome of interest not measured, non-randomized intervention, or limited sample. The resulting 29 reports were scrutinized, with 19 not meeting the protocol criteria [4,5,6,7,8,9,10,11,12,13,14,15,16,17,18,19,20,21,22]. Reasons for exclusion are listed in Supplementary Table S3. Ten trials or analyses were finally retained [23,24,25,26,27,28,29,30,31,32]. Figure 1 shows the study-selection flow chart.

### 3.2. Trial Characteristics

The retained reports covered three macro-areas: antiplatelet therapy (six trials), percutaneous coronary intervention (PCI) strategies (two trials) and beta-blocker treatment (two trials) [23,24,25,26,27,28,29,30,31,32]. Table 1 summarizes their main characteristics. Briefly, the number of randomized patients ranged from 656 in 4D-ACS to 8438 in REBOOT-CNIC; mean follow-up ranged from one year (six trials) to 3.7 years (REBOOT-CNIC); most patients presented ST- or non-ST-elevation MI (but also unstable angina), were between 60 and 67 years of age, and were treated by PCI on top of medical therapy; notable exceptions were TACSI-trial patients treated by coronary-artery bypass grafting (CABG), and FIRE-trial patients, who were older than 74 years (mean 81). The FIRE analysis stratified outcomes by renal function. The primary endpoint in six trials was the composite of all-cause or cardiac death and nonfatal ischemic events (including revascularization in most); the combination of ischemic and bleeding events in two trials; and the combination of death, MI and heart failure hospitalization or malignant arrhythmias in two trials. Safety endpoints included major or major plus clinically relevant nonmajor bleeds in seven trials; four trials alternatively or additionally assessed contrast-associated kidney injury, procedural complications, heart failure, or malignant arrhythmias.

### 3.3. Risk of Bias of the Included Trials

**Figure 2:**
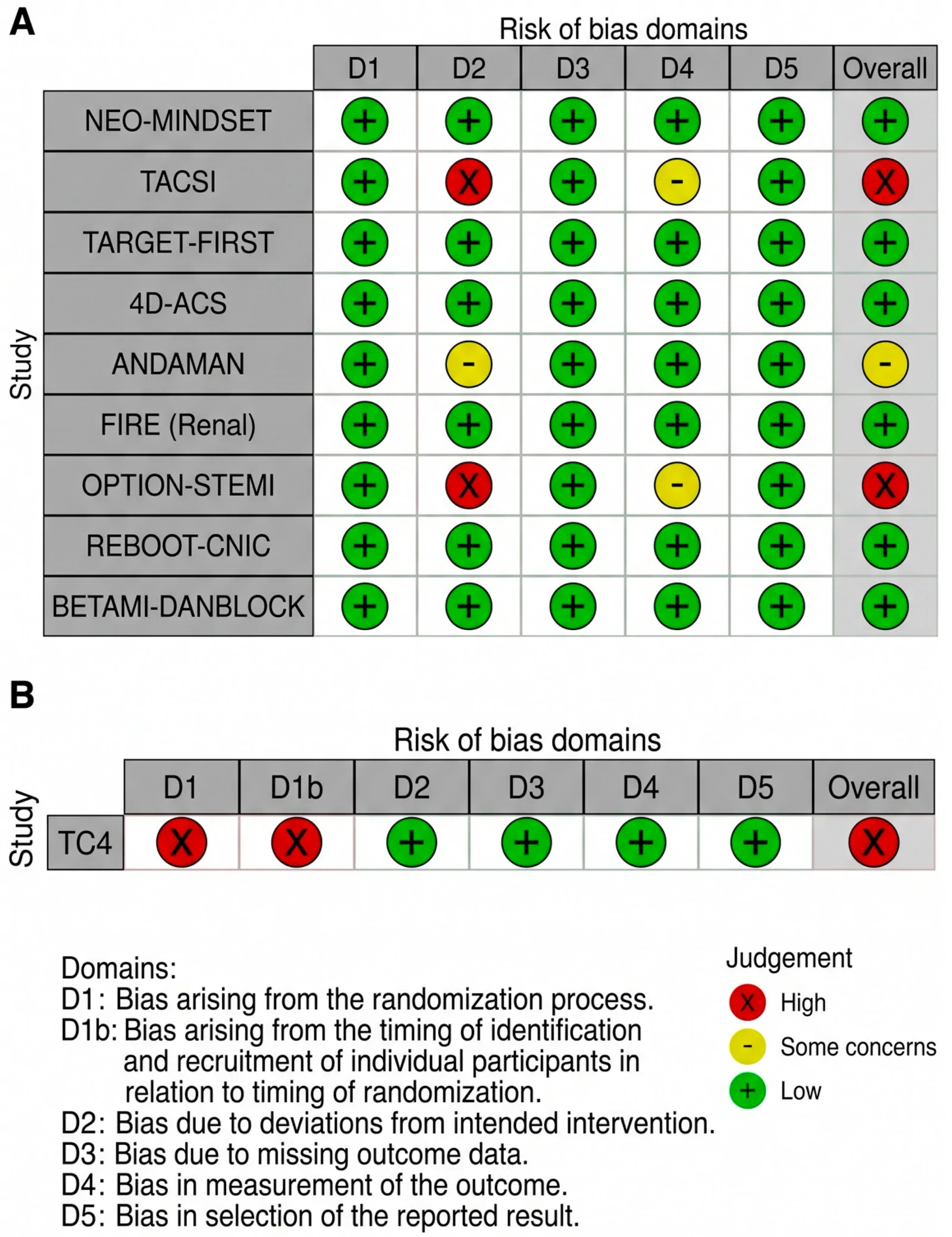
Risk of bias of the included reports. Panel (**A**): Individually randomized trials. Panel (**B**): TC4 cluster-randomized trial with the additional D1b domain.

Overall, the risk of bias was assessed as low in six trials, of some concern in one, and high in three. Assessment of each domain (allocation, deviations, missingness, measures and reporting) is shown in Figure 2; details are provided in Supplementary Table S4.

### 3.4. Main Findings of the Included Studies

#### 3.4.1. Duration of Dual Antiplatelet Therapy (DAPT) After PCI or CABG

Four open-label trials compared different DAPT durations in ACS patients undergoing revascularization.

In the Brazilian NEO-MINDSET trial of 3410 patients undergoing PCI, very early P2Y12-inhibitor monotherapy (within 4 days of hospitalization) failed to show non-inferiority compared to 12-month DAPT, with a hazard ratio (HR) for the primary endpoint (death, MI, stroke, urgent target vessel revascularization) of 1.28 (95% confidence interval [CI] 0.98–1.68) and absolute event rates of 7.0% vs. 5.5%; major or clinically relevant nonmajor bleeding on the other hand was significantly reduced by very early antiplatelet monotherapy (2.0% vs. 4.9%; HR 0.40, 95% CI 0.26–0.59) [23].

**Figure 3:**
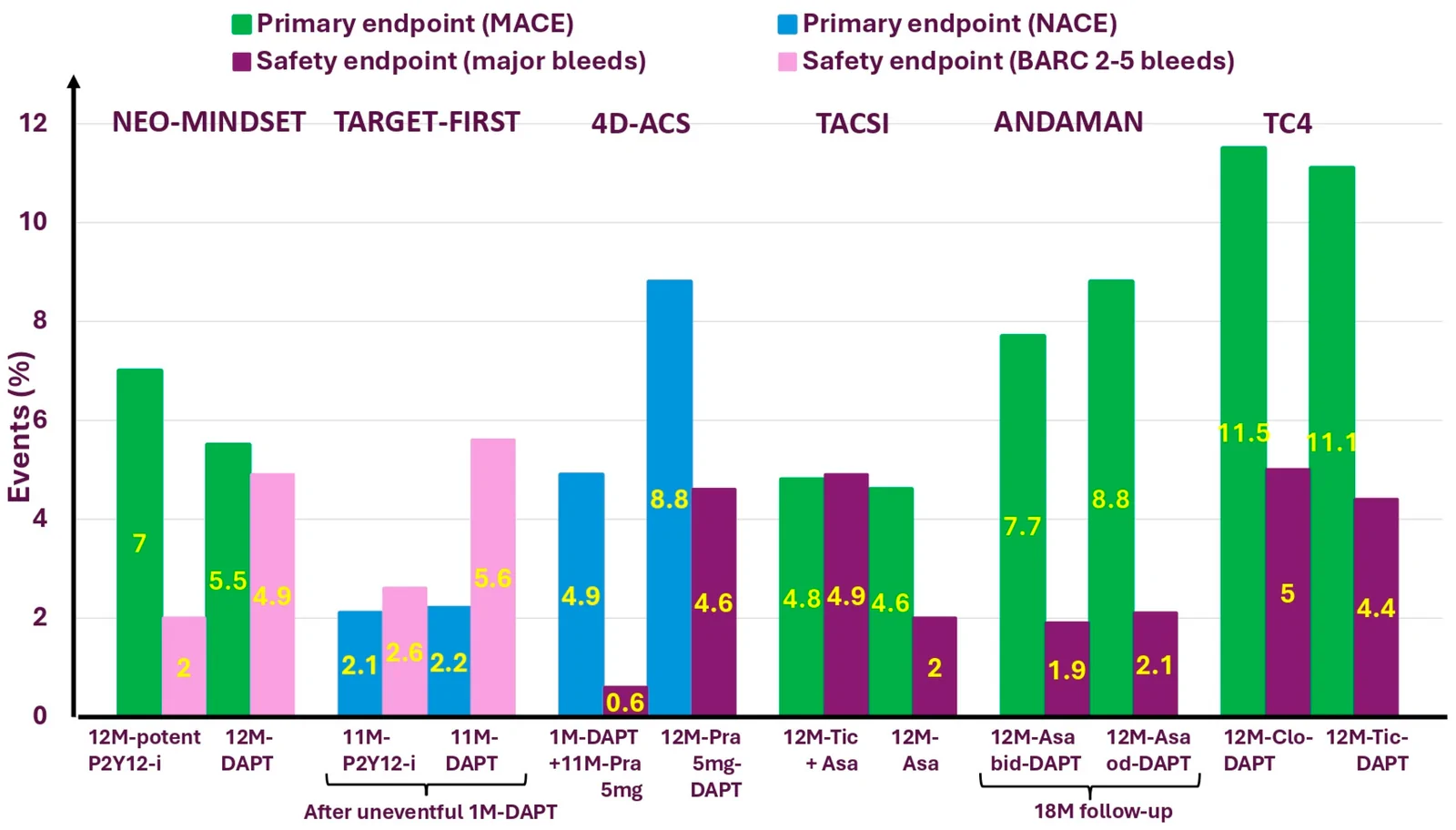
Events by antiplatelet strategy in six recent ACS trials over each trial duration [23,24,25,26,27,28]. NACE in TARGET-FIRST include BARC 3-5; NACE in 4D-ACS include BARC 2-5. ACS, acute coronary syndrome; ASA, aspirin; BARC, Bleeding Academic Research Consortium; CLO, clopidogrel; DAPT, dual antiplatelet therapy; i, inhibitor; M, month; MACE, major adverse cardiovascular events; NACE, net adverse cardiovascular events; PRA, prasugrel.

The European TARGET-FIRST trial of 1942 patients and the East Asian 4D-ACS trial of 656 patients both reported significantly lower bleeding rates without apparent loss of anti-ischemic protection with one-month full-dose DAPT followed by single antiplatelet therapy (SAPT), compared to 12-month DAPT, among low ischemic risk ACS patients undergoing PCI [25,26]. Reduced bleeding rates were driven by clinically relevant nonmajor bleeds in TARGET-FIRST and by major bleeds in 4D-ACS (Table 1 and Figure 3). Also, SAPT was achieved by aspirin cessation, so any effects of mid/long-term aspirin-based SAPT remain undefined [33].

The European TACSI trial enrolled 2201 ACS patients revascularized by CABG [24]. In this setting, where no prothrombotic intracoronary device is used [34], 12-month ticagrelor-based DAPT compared to very early aspirin monotherapy increased major bleeds (4.9% vs. 2.0%; HR 2.50, 95% CI 1.52–4.11) without apparent protection against a composite of death, MI, stroke, or repeat coronary revascularization (4.8% vs. 4.6%; HR 1.06, 95% CI 0.72–1.56) [24]. Most TACSI patients (90%) presented non-ST-elevation MI or unstable angina; also, the greater non-adherence in the DAPT arm compared to the aspirin arm (36% vs. 21%) and lack of a dedicated blinded adjudication committee may have biased the results.

#### 3.4.2. Different 12-Month DAPT Regimens

Two open-label ACS-PCI trials addressed this topic.

In the French ANDAMAN trial, conducted in 2484 ACS patients deemed at high risk of aspirin “resistance”, 100 mg twice-daily aspirin was compared to 100 mg once-daily aspirin (both on top of a P2Y12 inhibitor for a recommended 12-month period) [27]. High risk of aspirin resistance was defined by concomitant diabetes mellitus (in 77%), body mass index >26.9 Kg/m^2^, increased waist circumference, or onset of the index event while on aspirin. Thus, the population was at relatively high ischemic risk. Early revascularization by PCI was performed in 93%. Twice- versus once-daily aspirin did not yield significant differences in major ischemic (7.7% vs. 8.8%; HR 0.90, 95% CI 0.69–1.19) or major bleeding events (1.9% vs. 2.1%; HR 0.88, 95% CI 0.50–1.55) at the 1.5-year follow-up [27].

The Canadian, cluster-randomized TC4 trial reported no significant differences in 1-year major outcomes among 1005 ACS-PCI patients randomized to clopidogrel-DAPT or ticagrelor-DAPT [28]. In this single-center trial, 37% of patients had unstable angina; the clopidogrel loading dose was 300 (not 600) mg, and treatment allocation was by time-clusters, with randomization at the time of the first 2-month exposure, followed by simple alternation of subsequent 2-month assignments [28]. The incompletely concealed allocation may have introduced bias and may explain imbalances in the baseline characteristics of the treated groups. Major adverse cardiovascular event rates were notably higher compared to other contemporary trials.

The main findings from the six recent ACS trials on antiplatelet therapy are summarized in Figure 3.

#### 3.4.3. PCI Strategies

A prespecified trial analysis and an additional trial reported results of different PCI strategies in ACS patients.

**Figure 4:**
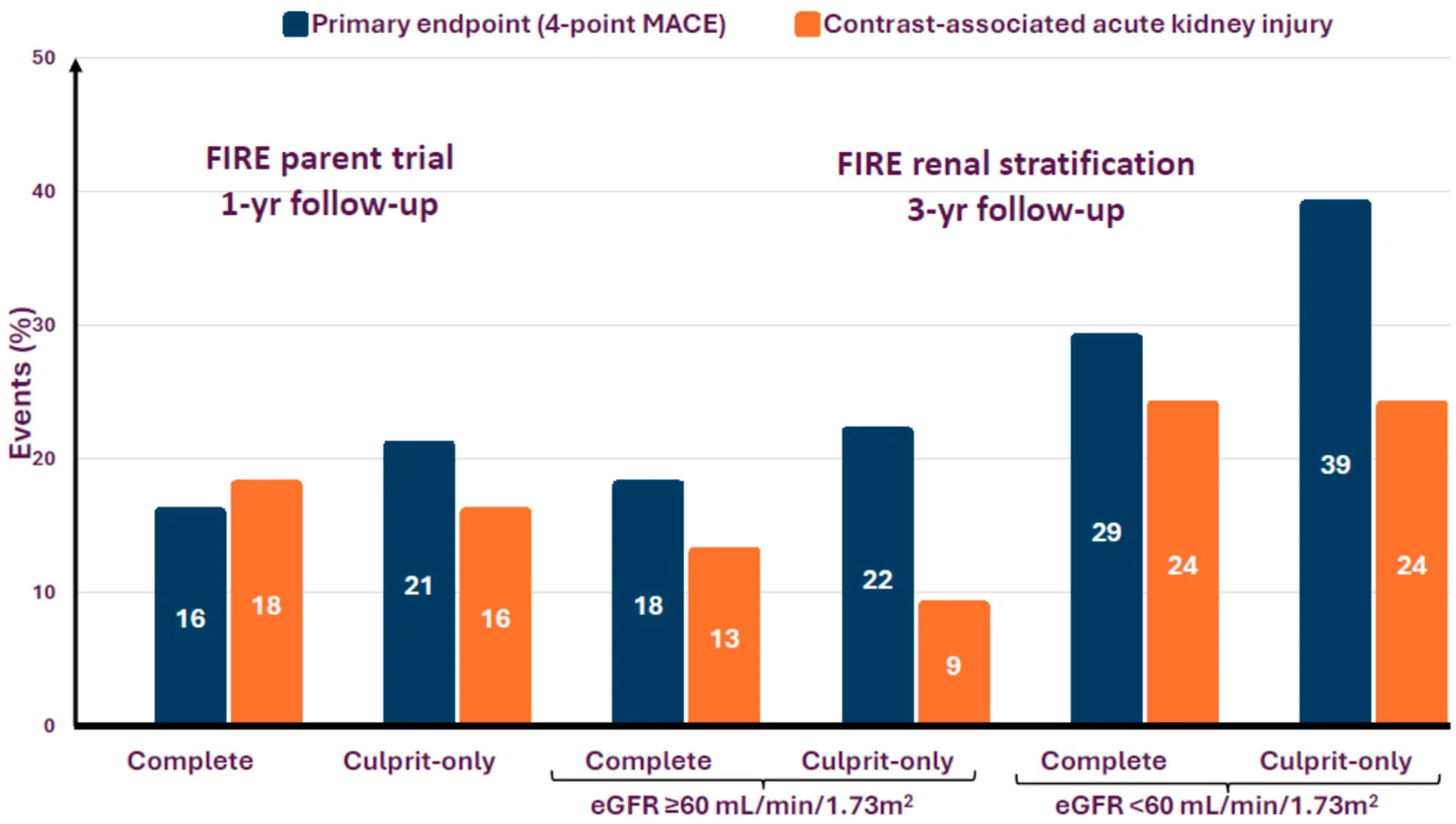
Events in the main FIRE trial at 1-year follow-up (left) and in the analysis by renal function strata at 3 years (right) [29,35]. MACE, major adverse cardiovascular events; eGFR, estimated glomerular filtration rate.

The multinational, open-label FIRE trial of 1445 ACS patients older than 74 years with multivessel disease had reported significantly lower 1-year rates of death, MI, stroke or ischemia-driven revascularization after physiology-guided complete revascularization compared to culprit-lesion-only revascularization (15.7% vs. 21.0%, *p* = 0.01) [35]. The prespecified analysis stratified by renal function (retained by the present review) confirmed the reported benefits of complete compared to culprit-only revascularization at a 3-year follow-up, regardless of renal function stratum, driven mainly by reduced cardiovascular mortality and ischemia-driven revascularizations [29]. Figure 4 summarizes the 1-year original FIRE findings as well as the 3-year findings by renal status.

In the open-label OPTION-STEMI trial [30], 994 patients with ST-elevation MI and multivessel disease, who had undergone successful primary PCI of the culprit lesion, were randomized to either immediate complete revascularization or to slightly delayed (pre-discharge) complete revascularization. The pre-discharge complete procedure was performed at a median of 3 days after primary PCI. For non-culprit lesions with visual stenoses of 50–69%, the protocol recommended revascularization guided by fractional flow reserve. Of 1303 non-culprit lesions, 63% in the immediate arm and 55% in the pre-discharge arm underwent revascularization. The primary endpoint at 1 year (death, MI, unplanned revascularization) did not reach non-inferiority for the immediate compared to the in-hospital staged procedure: 13% vs. 11% (HR 1.24, 95% CI 0.86–1.79; *p* = 0.24 for non-inferiority). There was a trend favoring pre-discharge complete revascularization in patients with Killip class II and III (23% vs. 13%; HR 1.79, 95% CI 1.05–3.05), or older than 64 years (19% vs. 13%; HR 1.49, 95% CI 0.97–2.29) [30]. In-hospital and 1-year adverse events did not differ significantly between treatment arms.

#### 3.4.4. Beta-Blocker Treatment

Beta-blocker treatment was compared to no beta-blocker treatment in two large, open-label trials retained by the present review. In REBOOT-CNIC and BETAMI-DANBLOCK, over 14,000 acute MI patients with a left ventricular ejection fraction (LVEF) above or equal to 40% were enrolled and followed for ≥3.5 years [31,32], thus covering both the acute and chronic phases after MI [36].

While the Spanish–Italian REBOOT-CNIC trial of 8438 patients reported no significant differences in the rates of a 3-item primary endpoint with or without beta-blocker treatment (2.25% vs. 2.17% patient-years; HR 1.04, 95% CI 0.89-1.22), the Danish–Norwegian BETAMI-DANBLOCK trial of 5574 patients reported significantly lower rates of the 6-item primary endpoint with beta-blocker treatment (14.2% vs. 16.3% over a median follow-up of 3.5 years; HR 0.85, 95% CI 0.75-0.98) [31,32]. The lower LVEF threshold for enrolment was 41% in REBOOT-CNIC and 40% in BETAMI-DANBLOCK. The primary endpoint comprised death, heart failure hospitalization and new MI in REBOOT-CNIC, and, additionally, unplanned revascularization, ischemic stroke and malignant ventricular arrhythmias in BETAMI-DANBLOCK [31,32].

## 4. Discussion

The ten reports identified by the present scoping review have implications for the daily management of ACS patients. Their novelty and clinical impact are best assessed in the context of the existing literature and of specific trial characteristics.

### 4.1. Antiplatelet Therapy

The default duration of DAPT following ACS is debated [37]. The 2023 European guidelines recommend 12-month DAPT as the default duration (class I, level of evidence A), with consideration of shorter durations for high-bleeding-risk patients (1 month: class IIb, level of evidence B) or low ischemic-risk patients (3–6 months: class IIa, level of evidence A) [38]. TARGET-FIRST and 4D-ACS showed enhanced safety with 1-month compared to 12-month DAPT regimens, without apparent loss of anti-ischemic protection [25,26]. Both trials enrolled relatively low ischemic-risk patients: TARGET-FIRST enrolled those who had already completed a first month without ischemic or bleeding complications, and 4D-ACS enrolled East Asian patients whose rates of ischemic events, on average, are reported to be lower compared to Caucasians [39,40]. Thus, generalizability of the two trial findings to higher ischemic-risk patients requires caution. Also, 4D-ACS used an Asian-approved prasugrel dosing of 5 mg daily in the 12-month DAPT arm and during the 11-month prasugrel monotherapy period (not the 10 mg routinely-approved by the European Medicines Agency and Food and Drug Administration [41]). In the East Asian 4D-ACS trial, but not in the European TARGET-FIRST, major bleeds increased with longer compared to shorter DAPT, consistent with more frequent and more severe bleeding events among East Asians compared to Caucasians receiving antithrombotic therapy [39,40]; in TARGET-FIRST, only Bleeding Academic Research Consortium type-2 bleeds were increased with prolonged DAPT (Table 1). The comparator DAPT duration of 11 or 12 months in the two trials prevents ascertaining potential benefits of intermediate DAPT durations (e.g., 3–6–9 months) compared to one-month DAPT. While the data of TARGET-FIRST and 4D-ACS should be interpreted with caution and in the context of each trial’s characteristics, the findings support consideration of DAPT durations shorter than 12 months in selected low ischemic-risk and/or high bleeding-risk patients.

The NEO-MINDSET trial failed to demonstrate non-inferiority of very early SAPT with a potent P2Y12 inhibitor compared to 12-month DAPT, with a HR for the primary endpoint of 1.28 (95% CI 0.98–1.68). Thus, the trial did not meet the prespecified criterion for non-inferiority with a point estimate favoring DAPT. The event rate was driven by all components of the primary endpoint, occurring mainly in the first 4 weeks after PCI [23]. In a very recent trial published after the search dates of the present review, 2216 Japanese ST-elevation MI patients undergoing primary PCI were randomized to either low-dose prasugrel monotherapy (20 mg loading dose followed by 3.75 mg daily) or low-dose prasugrel-based 12-month DAPT; although major bleeding was significantly reduced with monotherapy (5.6% vs. 8.4%), the 1-year primary outcome of death, MI or stroke occurred in 11% versus 8.5% (*p* = 0.41 for non-inferiority) [42]. Overall, the available data, in line with the 2023 ESC guidelines [38], do not support immediate antiplatelet monotherapy after ACS-PCI.

In the TACSI trial of ACS patients treated with CABG, ticagrelor-based DAPT was found to increase 1-year major bleeding without reducing ischemic event rates [24]. A previous individual patient meta-analysis of 871 CABG patients (44% with ACS at presentation) had reported superiority of ticagrelor-based DAPT over aspirin alone for vein graft patency at 3–12 month follow-up, without information on clinical outcomes [43]. The TACSI trial data—although likely influencing future ACS guidelines—should be interpreted with some caution given the high risk-of-bias concern and limited power for ischemic events. Current European guidelines support 12-month DAPT in ACS patients undergoing CABG (class I, level of evidence C) [38].

In the ANDAMAN trial, ischemic and bleeding outcomes at 1.5 years did not differ significantly in ACS patients deemed at high risk of aspirin “resistance” randomized to once- or twice-daily aspirin (on top of a P2Y12 inhibitor for the first 12 months) [27]. Previous ex vivo studies had documented more complete and longer-lasting platelet inhibition with twice-daily compared to once-daily aspirin monotherapy among non-ACS patients with diabetes mellitus or among higher body-mass individuals [44,45]. The ANDAMAN trial suggests that, within a DAPT regimen, twice- versus once-daily aspirin dosing does not influence clinical outcomes.

The Canadian TC4 trial reported similar posterior probabilities of major ischemic and bleeding events among ACS-PCI patients receiving ticagrelor- or clopidogrel-based 12-month DAPT [28]. In 2009, the multicenter PLATO (Study of Platelet Inhibition and Patient Outcomes) trial of 18,624 ACS patients had shown superiority of ticagrelor- over clopidogrel-based DAPT in preventing major adverse cardiovascular events at 12 months, but with an opposite trend among the 1814 North American participants (interaction-*p* 0.05) [46]. Limitations of the single-center TC4 trial include: (i) a relatively small sample; (ii) blinding of randomization only at start of trial; (iii) baseline differences between treatment arms (e.g., in age and proportion of unstable angina versus MI) and in median peak troponin values; (iv) outcome data of North American patients in the PLATO trial used as informative prior for the prespecified primary Bayesian analysis; (v) administrative data for outcome assessment; and (vi) lack of an independent event-adjudication committee. Also, the rates of ischemic events were considerably higher in TC4 compared to other recent trials, partly explained by an older mean age (67 vs. 60–62 years, Table 1) and recruitment during the COVID-19 pandemic [28]. Overall, although ticagrelor- compared to clopidogrel-based DAPT may yield lesser benefits in North American ACS patients (related, for instance, to clopidogrel responsiveness [47], reported to be higher among Caucasians than among Asian populations [48]), this topic remains debated.

### 4.2. PCI Strategies

The 3-year follow-up of the FIRE trial with stratification by renal function [29] confirmed the findings reported in the parent trial at one year, namely, that complete versus culprit-only revascularization in multivessel ACS patients older than 74 years can significantly reduce ischemic events (mainly cardiovascular mortality and ischemia-driven revascularizations) during follow-up [35]. The findings support complete physiology-guided revascularization across renal function strata in this elderly population [29,41].

Whether revascularization of non-culprit lesions in ST-elevation MI patients is best performed at the time of primary PCI or pre-discharge or at a later time is debated [38]. The OPTION-STEMI trial failed to demonstrate non-inferiority of immediate compared to pre-discharge complete revascularization, with a HR of 1.24 (95% CI 0.86–1.79) [30]. Although the trend favored pre-discharge timing particularly in specific groups, the data should not be interpreted as proof of superiority of the pre-discharge strategy. The 2023 European guidelines recommend complete revascularization for ST-elevation MI patients with multivessel disease, either immediately (excepting cardiogenic shock) or within 45 days, with a similar strength of recommendation (class I, level of evidence A), and with choices based on patient and disease characteristics (class I, level of evidence B) [38]. Thus, in line with the 2023 guidelines, the OPTION-STEMI data and recent meta-analyses suggest individualized decisions with regards to timing [30,49].

### 4.3. Beta-Blocker Treatment

In patients with an LVEF above or equal to 40% after MI, beta-blocker versus no beta-blocker treatment showed mixed effects in the REBOOT-CNIC and BETAMI-DANBLOCK trials [31,32]. In a landmark analysis, the REBOOT-CNIC investigators confirmed the neutral findings, both in the initial 12-month period and in the following chronic coronary syndrome period [10]. A subsequent individual patient data meta-analysis of four trials, including REBOOT-CNIC and BETAMI-DANBLOCK, focused on the 1885-patient subgroup with mildly reduced LVEF (40–49%), showing a significant reduction in the composite of death–MI–heart failure by beta-blocker therapy: 3.26% patient-years vs. 4.30% patient-years, *p* = 0.031, thereby extending the known benefits of beta-blockers in patients with an LVEF <40% to those with an LVEF after MI of 40–49% [50]. Conversely, another subsequent individual patient data meta-analysis of five trials, focusing on 17,801 patients with LVEF ≥50% after MI, showed non-significant differences in the composite or individual rates of death, MI or heart failure by beta-blocker therapy [51]. Consistent with the above data, the 2026 European heart failure guidelines include beta-blockers within the foundational medical therapy of symptomatic patients with LVEF up to 49% (class I, level of evidence A) [52].

**Figure 5:**
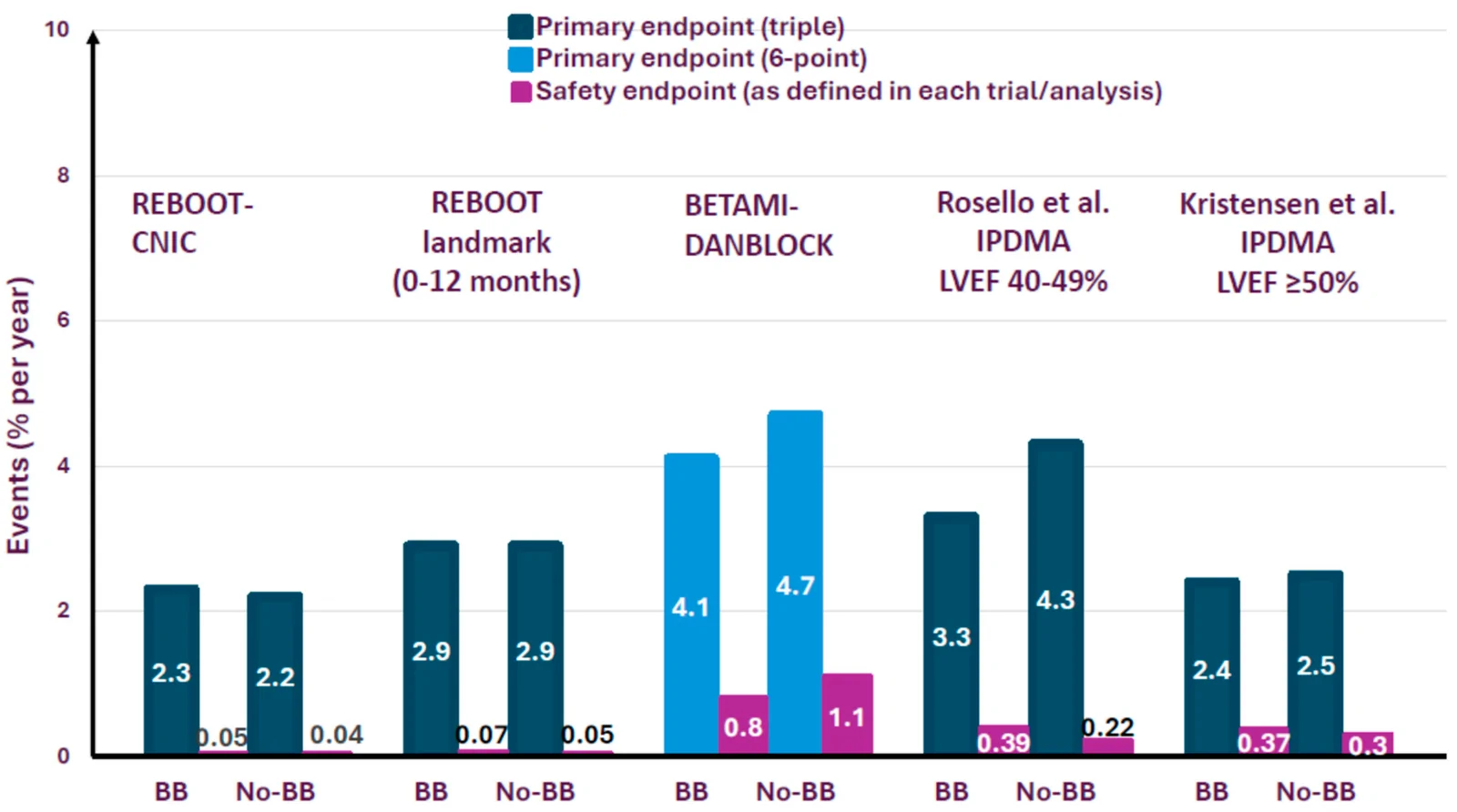
Annualized event rates after myocardial infarction by beta-blocker treatment and by LVEF [10,31,32,50,51]. The BETAMI-DANBLOCK safety endpoint refers to the first 30 days after randomization; the annualized BETAMI-DANBLOCK primary-endpoint estimates are a rough approximation based on median follow-up. BB, beta-blocker treatment; IPDMA, individual patient-data meta-analysis; LVEF, left ventricular ejection fraction.

The main findings of REBOOT-CNIC, BETAMI-DANBLOCK, and the two meta-analyses mentioned above are summarized in Figure 5.

### 4.4. Study Limitations

A number of limitations of the present review should be acknowledged. Although a prespecified protocol was followed, formal protocol registration was not carried out. The time frame of the review spans from 1 January 2025 to 29 March 2026. Within this time frame, relevant articles may have been overlooked, although the defined search criteria and list of excluded studies should allow replicability. Analysis by type of ACS (ST-elevation MI, non-ST-elevation MI, or unstable angina) was not performed, given inevitable loss of statistical power. The scoping nature of the review prevented synthesis of the results by summary measures.

## 5. Conclusions and Next Steps

The present scoping review of recent clinical outcome trials in ACS patients informs decisions on antiplatelet therapy, percutaneous coronary interventions and beta-blocker treatment that commonly arise in daily practice. Broadly, the results favor (i) cautious shortening of dual antiplatelet therapy in selected, low ischemic-risk ACS patients, (ii) complete revascularization in appropriately selected patients, with timing individualized to clinical presentation, lesion characteristics, and patient stability, and (iii) refraining from routine beta-blocker treatment in patients with an LVEF after MI ≥ 50%.

Prevention and management of coronary syndromes remain challenging. Future advances will likely benefit from greater understanding of vulnerable plaque characteristics [53], of biomarker-led information [54], and of the mechanisms of ACS in under-represented trial populations, such as women [55,56] or those without traditional cardiovascular risk factors. Universal access to guideline-directed care remains another powerful unmet need [57].

## Figures and Tables

**Table 1:** Main characteristics and findings of the included trials.

Trial, Design, Funding	N, Patients, Setting	Age, Sex %	n—Active Treatment	n—Control Treatment	FU	Primary Endpoint (PEP) and Safety Endpoint (SEP)	Main Findings
**NEO-MINDSET** [23] - Open-label - PEP NI (2.5% margin) and SEP SUP - Academic	3410 Brazilian ACS receiving DES-PCI: - 2119 STEMI - 1039 NSTEMI - 252 UA	- 60 ± 11 yrs - Male 70%, female 30%	1712—12-mo potent P2Y12-i monotherapy	1698—12-mo aspirin 100 mg od + potent P2Y12-i	1 yr	PEP: death, MI, stroke, or urgent target vessel revascularization SEP: BARC 2, 3, 5 bleeds	SAPT vs. DAPT: PEP: not NI 7.0% vs. 5.5%, HR 1.28, 95% CI 0.98–1.68 SEP: 2.0% vs. 4.9%, HR 0.40, 95% CI 0.26–0.59 (driven by BARC 2-3)
**TACSI** [24] - Open-label - SUP for PEP - Academic	2201 North European CABG-ACS: - 10% STEMI - 57% NSTEMI - 33% UA	- 66 ± 9 yrs - Male 86%, female 14%	1104—ticagrelor 90 mg bid + aspirin 75–100 mg od	1097—aspirin 75–160 mg od	1 yr	PEP: death, MI, stroke, or repeat revascularization SEP: major (hospitalized) bleeding	T + A vs. A alone: PEP: 4.8% vs. 4.6%, HR 1.06, 95% CI 0.72–1.56. SEP: 4.9% vs. 2.0%, HR 2.50, 95% CI 1.52–4.11
**TARGET-FIRST** [25] - Open-label - NI for NACE (1.5% margin) - SUP for SEP - MicroPort	1942 European ACS undergoing PCI: - 979 STEMI - 963 NSTEMI	- 61 ± 11 yrs - Male 78%, female 22%	961—11-mo P2Y12-i after first uneventful month of DAPT	981—11-mo DAPT after first uneventful month of DAPT	1 yr	PEP: NACE = death, MI, stent thrombosis, stroke, or major (BARC 3 or 5) bleeds SEP: BARC 2, 3, or 5 bleeds	1-mo vs. 12-mo DAPT: PEP: 2.1% vs. 2.2%; *p* = 0.02 for NI SEP: 2.6% vs. 5.6%, HR 0.46, 95% CI 0.29–0.75, *p* = 0.002 (driven by BARC 2 bleeds)
**4D-ACS** [26] - Open-label - NI and SUP for NACE - Academic	656 S. Korean ACS receiving drug-coated stents: - 215 STEMI - 201 NSTEMI - 240 UA	- 61 ± 10 yrs - Male 82%, female 18%	328—1-mo aspirin 100 mg od + prasugrel 10 mg od followed by 11-mo prasugrel 5 mg od	328—12-mo aspirin 100 mg od + prasugrel 5 mg od	1 yr	PEP: NACE = death, MI, stroke, ischemia-driven target vessel revascularization, or BARC 2-5 bleeds SEP: major (BARC 3-5) bleeds	1-mo full-dose DAPT followed by moderate SAPT vs. 12-mo moderate DAPT: NACE: 4.9% vs. 8.8%, HR 0.51, 95% CI 0.27–0.95, *p* = 0.034 (driven by BARC 3-5 bleeds) SEP: 0.6% vs. 4.6%, HR 0.13, 95% CI 0.03–0.58, *p* = 0.007
**ANDAMAN** [27] - Open-label - SUP for MACE - Academic	2484 French ACS at high risk of aspirin resistance: - 1377 STEMI - 1107 NSTEMI	- 62 ± 11 yrs - Male 80%, female 20%	1228—12-mo aspirin 100 mg bid on top of P2Y12-i, then SAPT	1256—12-mo aspirin 100 mg od on top of P2Y12-i, then SAPT	1.5 yrs	PEP: MACE = death, MI, stroke, urgent revascularization, ST, other arterial thrombosis SEP: BARC 3-5 bleeds	Bid vs. od aspirin: MACE: 7.7% vs. 8.8%, HR 0.90, 95% CI 0.69–1.19, *p* = 0.42 SEP: 1.9% vs. 2.1%, HR 0.88, 95% CI 0.50–1.55
**TC4** [28] - Open-label - Time-clustered - Posterior probability of SUP for PEP - Academic	1005 Canadian ACS receiving PCI: - 21% STEMI - 42% NSTEMI - 37% UA	- 67 ± 11 yrs - Male 75%, female 25%	450—12-mo T 180 mg LD, then 90 mg bid + A 325 mg LD, then 81 mg od	555—12-mo C 300 mg LD, then 75 mg od + A 325 mg LD, then 81 mg od	1 yr	PEP: MACE = death, MI, or ischemic stroke SEP: major (hospitalized) bleeding	T vs. C: MACE: 11.1% vs. 11.5%, RR 0.95, 95% credible interval 0.67–1.35 SEP: 4.4% vs. 5.0%, RR 0.89, 95% credible interval 0.52–1.52
**Prespecified FIRE** **analysis by renal strata** [29] - Open-label - SUP for PEP and SEP, in parent trial - Academic	1445 Italian, Polish and Spanish MI ≥ 75 yrs with MVD: - 662 eGFR < 60 - 783 eGFR ≥ 60 35% STEMI	- 81 ± 4 yrs - Male 63%, female 37%	720—complete revascularization: - 330 eGFR < 60 - 390 eGFR ≥ 60	725—culprit-only revascularization: - 332 eGFR < 60 - 393 eGFR ≥ 60	3 yrs	PEP: death, MI, stroke, or ischemia-driven revascularization Parent trial at 1 year: complete vs. culprit: 15.7% vs. 21%, HR 0.73, 95% CI 0.57–0.93, *p* = 0.01 SEP: contrast-AKI, stroke, or BARC 3-5 Parent trial: complete vs. culprit: 22.5% vs. 20.4%, HR 1.11, 95% CI 0.89–1.37	Complete vs. culprit: PEP: eGFR < 60 HR 0.68, 95% CI 0.52–0.89 eGFR ≥ 60 HR 0.80, 95% CI 0.59–1.10 *p-int >* 0.42 Stroke and BARC 3-5: NS *p-int* for treatment-by-renal category Contrast-AKI: eGFR <60 23.9% vs. 23.8%, *p* = 0.97 eGFR ≥ 60 12.8% vs. 9.4%, *p* = 0.13
**OPTION-STEMI** [30] - Open-label - NI for the PEP (margin < 1.42 for the upper HR boundary) - Boston Scientific	994 S. Korean STEMI with MVD, undergoing successful primary PCI of culprit lesion	- 66 (57–76 IQ) yrs - Male 79%, female 21%	498—immediate revascularization of non-culprit lesion(s)	496—staged revascularization of non-culprit lesions (on another day but before discharge)	1 yr	PEP: death, MI, unplanned revascularization SEP: procedural complications, contrast-induced nephropathy, clinical events during index admission	Immediate vs. staged: PEP: not NI 13% vs. 11%, HR 1.24 (95% CI 0.86–1.79, *p* = 0.24) SEP: no significant differences for all comparisons
**REBOOT-CNIC** [31] - Open-label - SUP for PEP - Academic	8438 Spanish/Italian ACS with LVEF > 40%: - 4296 STEMI - 4142 NSTEMI	- 61 ± 11 yrs - Male 81%, female 19%	4207—beta-blocker treatment	4231—no beta-blocker treatment	3.7 yrs	PEP: death, re-MI, or hospitalization for heart failure SEP: hospitalization for symptomatic advanced AV block	BB vs. no BB: PEP: 22.5 vs. 21.7 per 1000 patient-yrs, HR 1.04, 95% CI 0.89–1.22 AV block: 0.5 vs. 0.4 per 1000 patient-yrs, HR 1.18, 95% CI 0.40–3.50
**BETAMI-DANBLOCK** [32] - Open-label - SUP for PEP - Academic & Novo Nordisk Foundation	5574 Norwegian/Danish AMI patients with LVEF ≥ 40%: - 48% STEMI - 52% NSTEMI	- 63 (55–71 IQ) yrs - Male 79%, female 21%	2783—beta-blocker treatment	2791—no beta-blocker treatment	3.5 yrs	PEP: death and MACE (MI, unplanned revascularization, ischemic stroke, HF, or MVA) SEP: death, MI, HF, or MVA within 30 days after randomization	BB vs. no BB: PEP: 14.2% vs. 16.3%, HR 0.85, 95% CI 0.75–0.98) SEP: 0.8% vs. 1.1%

Abbreviations: A, aspirin; ACS, acute coronary syndrome; AKI, acute kidney injury; AMI, acute myocardial infarction; AV, atrioventricular; BARC, Bleeding Academic Research Consortium; BB, beta-blocker; bid, twice daily; C, clopidogrel; CABG, coronary artery bypass grafting; DAPT, dual antiplatelet therapy; DES, drug-eluting stent; DM, diabetes mellitus; eGFR, estimated glomerular filtration rate (ml/min/1.73 m^2^); FU, follow-up; HF, heart failure; HR, hazard ratio; IQ, interquartile; LD, loading dose; LVEF, left ventricular ejection fraction; MACE, major adverse cardiovascular event; MI, myocardial infarction; mo, month; MVA, malignant ventricular arrhythmias; MVD, multivessel disease; NACE, net adverse clinical events; NI, non-inferiority; NSTEMI, non-ST-elevation myocardial infarction; od, once daily; P2Y12-i, P2Y12 inhibitor; PCI, percutaneous coronary intervention; PEP, primary endpoint; RR, relative risk; SAPT, single antiplatelet therapy; SEP, safety endpoint; ST, stent thrombosis; STEMI, ST-elevation myocardial infarction; SUP, superiority; T, ticagrelor; UA, unstable angina; y, years. Trial acronyms: ANDAMAN, Aspirin with a Novel twice-a-day administration in patients with Diabetes mellitus after Acute coronary syndrome to Minimize recurrence of Acute ischemic events or New urgent revascularization; BETAMI, Norwegian Beta-Blocker Treatment after Acute Myocardial Infarction in Revascularized Patients without Reduced Left Ventricular Ejection Fraction; DANBLOCK, Danish Trial of Beta-Blocker Therapy after Myocardial Infarction without Heart Failure; 4D-ACS, Duration of DAPT followed by Dose reduction after Implantation of DCS in ACS patients; FIRE, Functional Assessment in Elderly MI Patients with Multivessel Disease; NEO-MINDSET, percutaNEOus coronary intervention followed by Monotherapy INstead of Dual antiplatelet therapy in the SETting of acute coronary syndromes; OPTION-STEMI, OPTImal timing of fractional flow reserve-guided complete revascularizatiON for non-infarct-related artery in ST-segment Elevation Myocardial Infarction with multivessel disease; REBOOT-CNIC, Treatment with Beta-Blockers after Myocardial Infarction without Reduced Ejection Fraction—Centro Nacional de Investigaciones Cardiovasculares Carlos III; TACSI, Dual Antiplatelet Therapy with Ticagrelor and ASA vs. ASA Only after Isolated Coronary Artery Bypass Grafting in Patients with Acute Coronary Syndrome; TARGET-FIRST, Evaluation of a Modified Anti-Platelet Therapy Associated With Low-dose DES Firehawk in Acute Myocardial Infarction Patients Treated With Complete Revascularization Strategy; TC-4, Ticagrelor compared to Clopidogrel in Acute Coronary Syndromes.

## Data Availability

The original data reported in this article are available in the listed PubMed/MEDLINE citations.

## Supplementary Materials

The following supporting information can be downloaded at https://www.mdpi.com/article/10.3390/jcm15218704/s1: Table S1: PubMed/MEDLINE search details; Table S2: PRISMA-scoping review checklist; Table S3: Excluded reports [4,5,6,7,8,9,10,11,12,13,14,15,16,17,18,19,20,21,22]; Table S4: Details of risk of bias assessment.

## Abbreviations

The following abbreviations are used in the text of this manuscript:

ACS	Acute coronary syndrome
CABG	Coronary artery bypass graft
DAPT	Dual antiplatelet therapy
IPDMA	Individual patient-data meta-analysis
LVEF	Left ventricular ejection fraction
MI	Myocardial infarction
PCI	Percutaneous coronary intervention
PRISMA	Preferred Reporting Items for Systematic reviews and Meta-Analyses
RCT	Randomized controlled trial
SAPT	Single antiplatelet therapy
ScR	Scoping review
