# Supplementary material for: Focused Update on Randomized Trials in Acute Coronary Syndromes: A Scoping Review
Source: J Clin Med. 2026 Nov 7;15(21):8704. doi: 10.3390/jcm15218704 (PMC13764379; doi:10.3390/jcm15218704)
Supplement: Supplementary file 1 [file jcm-15-08704-s001.zip › jcm-4412219-supplementary.pdf]

Supplementary Appendix

Focused Update on Randomized Trials in Acute Coronary Syndromes: A Scoping Review

Andreotti et al.

Table of Content

Supplementary Table S1. PubMed/MEDLINE search details...S2

Supplementary Table S2. PRISMA-scoping review checklist...S3

Supplementary Table S3. Excluded reports..S5

Supplementary Table S4. Details of risk of bias assessment...S6

Supplementary Table S1. PubMed/MEDLINE search details

**Search strings**
Acute coronary syndrome and randomized and trial and 2025
Acute coronary syndrome and randomized and trial and 2026
Acute myocardial infarction and randomized and trial and 2025
Acute myocardial infarction and randomized and trial and 2026
**Automated filters for**
Randomized controlled trial
Patients ≥19 years of age
Full-text articles
English language
**Deduplication**
Based on DOI matching
**Search dates**
Inclusion from 1 January 2025 to 29 March 2026 (any eligible publication)
E-publications before 1 January 2025 excluded
**Other**
Manual retrieval from citation searches

Supplementary Table S2. PRISMA-scoping review checklist

SECTION	ITEM	PRISMA-ScR CHECKLIST ITEM	REPORTED ON PAGE #
**TITLE**			
Title	1	Identify the report as a scoping review.	1 of main text
**ABSTRACT**			
Structured summary	2	Provide a structured summary that includes (as applicable): background, objectives, eligibility criteria, sources of evidence, charting methods, results, and conclusions that relate to the review questions and objectives.	1 of main text
**INTRODUCTION**			
Rationale	3	Describe the rationale for the review in the context of what is already known. Explain why the review questions/objectives lend themselves to a scoping review approach.	2 of main text
Objectives	4	Provide an explicit statement of the questions and objectives being addressed with reference to their key elements (e.g., population or participants, concepts, and context) or other relevant key elements used to conceptualize the review questions and/or objectives.	2 of main text
**METHODS**			
Protocol and registration	5	Indicate whether a review protocol exists; state if and where it can be accessed (e.g., a Web address); and if available, provide registration information, including the registration number.	2 of main text
Eligibility criteria	6	Specify characteristics of the sources of evidence used as eligibility criteria (e.g., years considered, language, and publication status), and provide a rationale.	2 of main text
Information sources*	7	Describe all information sources in the search (e.g., databases with dates of coverage and contact with authors to identify additional sources), as well as the date the most recent search was executed.	2 of main text
Search	8	Present the full electronic search strategy for at least 1 database, including any limits used, such that it could be repeated.	2 of appendix
Selection of sources of evidence†	9	State the process for selecting sources of evidence (i.e., screening and eligibility) included in the scoping review.	2 of main text
Data charting process‡	10	Describe the methods of charting data from the included sources of evidence (e.g., calibrated forms or forms that have been tested by the team before their use, and whether data charting was done independently or in duplicate) and any processes for obtaining and confirming data from investigators.	2 of main text
Data items	11	List and define all variables for which data were sought and any assumptions and simplifications made.	2-3 of main text
Critical appraisal of individual sources of evidence§	12	If done, provide a rationale for conducting a critical appraisal of included sources of evidence; describe the methods used and how this information was used in any data synthesis (if appropriate).	3-4 of main text and 6-7 of apppendix

SECTION	ITEM	PRISMA-ScR CHECKLIST ITEM	REPORTED ON PAGE #
Synthesis of results	13	Describe the methods of handling and summarizing the data that were charted.	2-3 of main text
**RESULTS**			
Selection of sources of evidence	14	Give numbers of sources of evidence screened, assessed for eligibility, and included in the review, with reasons for exclusions at each stage, ideally using a flow diagram.	3 of main text and 5 of appendix
Characteristics of sources of evidence	15	For each source of evidence, present characteristics for which data were charted and provide the citations.	5 and 6 of main text
Critical appraisal within sources of evidence	16	If done, present data on critical appraisal of included sources of evidence (see item 12).	3-4 of main text and 6-7 of appendix
Results of individual sources of evidence	17	For each included source of evidence, present the relevant data that were charted that relate to the review questions and objectives.	7-10 of main text
Synthesis of results	18	Summarize and/or present the charting results as they relate to the review questions and objectives.	6-7 of main text
**DISCUSSION**			
Summary of evidence	19	Summarize the main results (including an overview of concepts, themes, and types of evidence available), link to the review questions and objectives, and consider the relevance to key groups.	10-12 of main text
Limitations	20	Discuss the limitations of the scoping review process.	12-13 of main text
Conclusions	21	Provide a general interpretation of the results with respect to the review questions and objectives, as well as potential implications and/or next steps.	13 of main text
**FUNDING**			
Funding	22	Describe sources of funding for the included sources of evidence, as well as sources of funding for the scoping review. Describe the role of the funders of the scoping review.	14 of main text

Supplementary Table S3. Excluded reports and reason for exclusion

STUDY DOI	REASON FOR EXCLUSION
doi: 10.1016/j.carrev.2025.03.001	*Post hoc* subgroup analysis
doi: 10.1016/j.jacc.2025.05.013	*Post hoc* subgroup analysis
doi: 10.1161/CIRCINTERVENTIONS.125.015902	*Post hoc* subgroup analysis
doi: 10.1016/j.jcin.2025.09.026	*Post hoc* subgroup analysis
doi: 10.1001/jamanetworkopen.2025.11297	*Post hoc* subgroup analysis
doi: 10.1001/jamanetworkopen.2024.61916	*Post hoc* subgroup analysis
doi: 10.1093/ehjcvp/pvag002	*Post hoc* landmark analysis
doi: 10.1056/NEJMoa2600440	Mixed population
doi: 10.1056/NEJMoa2601521	Mixed population
doi: 10.1016/j.jacc.2025.12.018	Comorbid shock
doi: 10.1136/bmj-2024-082945	Super-selected population
doi: 10.1016/j.annemergmed.2025.10.014	Outcome of interest not measured
doi: 10.1515/cclm-2024-0138	Outcome of interest not measured
doi: 10.1161/CIRCOUTCOMES.124.011441	Lack of parallel-treatment arms
doi: 10.1016/j.jcin.2025.01.434	Overlapping information
doi: 10.1093/eurheartj/ehaf673	Overlapping information
doi: 10.1056/NEJMoa2405922	First published before 2025
doi: 10.1056/NEJMoa2405923	First published before 2025
doi: 10.1016/j.jcin.2024.09.061	First published before 2025

Doi codes correspond to references 4-22 in the main text.

Supplementary Table S4. Details of risk of bias assessment

Trial	Low-risk domains	High risk / some concerns domain	Comments	Overall risk of bias
**NEO-MINDSET**	✓ Randomization process<br>✓ Deviations from intended interventions<br>✓ Missing outcome data<br>✓ Measurement of the outcome<br>✓ Selection of the reported result			Low risk
**TACSI**	✓ Randomization process<br>✓ Missing outcome data<br>✓ Selection of the reported result	✓ Deviations from intended interventions<br>✓ Measurement of the outcome	Asymmetric non-adherence to ticagrelor<br>Absence of blinded outcome adjudication	High risk
**TARGET-FIRST**	✓ Randomization process<br>✓ Deviations from intended interventions<br>✓ Missing outcome data<br>✓ Measurement of the outcome<br>✓ Selection of the reported result			Low risk
**4D-ACS**	✓ Randomization process<br>✓ Deviations from intended interventions<br>✓ Missing outcome data<br>✓ Measurement of the outcome<br>✓ Selection of the reported result			Low risk
**ANDAMAN**	✓ Randomization process<br>✓ Missing outcome data<br>✓ Measurement of the outcome<br>✓ Selection of the reported result	✓ Deviations from intended interventions	Compliance not formally assessed and tablet counts not collected	Some concerns
**TC4**	✓ Deviations from intended interventions<br>✓ Missing outcome data<br>✓ Measurement of the outcome	✓ Randomization process<br>✓ Timing of identification and recruitment of Individual participants in relation to timing of randomization<br>✓ Selection of the reported result	Allocation sequence after first cluster by simple alternation, not randomization<br>Baseline imbalances between ticagrelor and clopidogrel groups	High risk
**FIRE (Renal stratification)**	✓ Randomization process<br>✓ Deviations from intended interventions			Low risk

	✓ Missing outcome data<br>✓ Measurement of the outcome<br>✓ Selection of the reported result			
**OPTION-STEMI**	✓ Randomization process<br>✓ Missing outcome data<br>✓ Selection of the reported result	✓ Deviations from intended interventions<br>✓ Measurement of the outcome	Asymmetric crossover, exceeding the pre-specified 4% dropout rate<br>Unplanned revascularisation, a primary composite component, not adjudicated in a blinded manner	High risk
**REBOOT-CNIC**	✓ Randomization process<br>✓ Deviations from intended interventions<br>✓ Missing outcome data<br>✓ Measurement of the outcome<br>✓ Selection of the reported result			Low risk
**BETAMI-DANBLOCK**	✓ Randomization process<br>✓ Deviations from intended interventions<br>✓ Missing outcome data<br>✓ Measurement of the outcome<br>✓ Selection of the reported result			Low risk
